# Effects of Culture Medium Enrichment with Zinc on Astaxanthin Accumulation in a New Strain of the Microalga *Dysmorphococcus globosus*

**DOI:** 10.3390/plants13233338

**Published:** 2024-11-28

**Authors:** Zhaohui Zan, Xinxin Huang, Zahid Hussain, Moyu Zhong, Chenyang Hou, Maozhi Ren, Xiulan Xie

**Affiliations:** 1 Functional Plant Cultivation and Application Teams, Institute of Urban Agriculture, Chinese Academy of Agricultural Sciences, Chengdu 610000, Chinarenmaozhi01@caas.cn (M.R.); 2 School of Agricultural Sciences, Zhengzhou University, Zhengzhou 450052, China

**Keywords:** astaxanthin, zinc enrichment, microalgae, hidden starvation, one health, nutritional value, zinc adsorption capacity

## Abstract

High Zn^2+^ concentrations in microalgal cells that produce astaxanthin as a feed additive can reduce the symptoms of malnutrition in aquatic animals. Therefore, in this study, we analysed the effect of Zn^2+^ in the culture medium on the growth of a newly isolated microalgal strain *Dysmorphococcus globosus* ZY24. Zn^2+^ and white light stress altered the pigment content in microalgal cells. In addition, high Zn^2+^ concentrations in the culture medium altered cell morphology and chlorophyll fluorescence and also increased intracellular Zn^2+^ accumulation. Further, an optimal Zn^2+^ concentration in the culture medium promoted the synthesis of astaxanthin and other pigments. When the concentration of Zn^2+^ was 45.5 mg L^−1^, *Dysmorphococcus globosus* ZY24 produced 0.31 mg g^−1^ astaxanthin, whereas the total zinc content of the microalgae was 4337 mg kg^−1^. This study confirmed that microalgae have a high capacity for Zn^2+^ enrichment, providing a theoretical basis for studying Zn^2+^ enrichment in microalgae. Furthermore, Zn^2+^ supplementation to stimulate astaxanthin production in microalgae is a practical method to enhance their nutritional value.

## 1. Introduction

Fish farming has emerged as one of the most sustainable methods for providing humans with nutritious food. Indeed, aquaculture is rapidly becoming the primary source of edible proteins and is growing faster than any other food industry [1]. However, this industry is influenced by several interrelated factors, including culture environment, diet, and farmed stocks [2]. Enhancing fish farming conditions via appropriate and healthy diets is an important approach for improving fish health, growth, and welfare, as well as promoting feed utilisation and fish meat quality [3]. However, under the current situations associated with large-scale aquaculture in fisheries, it has become very difficult to feed fish with the right amount of nutrients [4]. Consequently, producing fish feed on large scales is the most essential requirement of the aquaculture sector. For instance, aquafeed consumption accounts for approximately 40 to 75% of the total operational cost in the aquaculture sector [5]. Fish feed directly affects fish quality and health, and ultimately impacts fish productional costs. Therefore, the use of cost-effective and eco-friendly technologies is necessary to mitigate these challenges and enhance sustainable aquaculture [6].

Zinc (Zn) is an essential trace element in living organisms; it is required for several physiological functions, including growth, development, reproduction, immunity, bone formation, and cell proliferation. Low Zn concentrations in the aquatic environment result in reduced fish growth [7]. Zn deficiency affects all aspects of the physiological and metabolic processes of fish, including their growth rates, feed intake, reproductive capacity, immune responses, and survival rates [8]. Therefore, supplementing fish diets with adequate Zn concentrations is necessary for normal fish growth.

Microalgae are ubiquitous photosynthetic microorganisms that inhabit aquatic environments and are vital primary producers in aquatic ecosystems. Microalgae play several roles in aquaculture by modifying the aquatic environment and serving as a food source. They have significant advantages, such as high versatility and differentiation, fast rate of growth, and survival under severe conditions [9]. Naturally categorised groups of algae are heterokont algae, green algae, red algae, blue-green algae, and charophytes; in detail, diatoms form nearly one-third of total algae species [10]. They are rich in various nutrients, including astaxanthin, β-carotene, proteins, and trace elements [11]. Furthermore, they can be cultivated on a large scale using biotechnological and other approaches, such as large-scale photobioreactors [12].

Unlike traditional crops, microalgae possess a unique cellular structure that enables them to accumulate high amounts of trace elements and bioactive compounds. For example, microalgae actively absorb heavy metals present in water bodies, and can thus be widely used for wastewater and sewage treatment [13]. Microalgae can grow in wastewater bodies, such as pig wastewater bodies, efficiently absorb nutrients, and synthesise several biological compounds, including proteins, carbohydrates, and fatty acids, without causing secondary pollution [14]. Wastewater bodies contain large amounts of Zn, which microalgae can accumulate and combine with biomolecules via metabolic processes, forming Zn-containing biological matter that can then be absorbed and used by the organism, and which promotes its healthy growth [15]. Additionally, through biosorption and biodegradation, microalgae can absorb a wide range of organic pollutants present in wastewater as energy sources for growth [16]. For example, Zhou et al. showed that *Arthrospira platensis* has an excellent Zn enrichment capacity [17]. Similarly, Liu et al. showed that in pig farm wastewater containing 1.85 mg L^−1^ Zn^2+^ and 1 mg L^−1^ Mn^2+^, *Chlorella* sp. removed approximately 86.72 and 42.74% of Zn^2+^ and Mn^2+^, respectively [18].

Carotenoids, including astaxanthin, lutein, zeaxanthin, canthaxanthin, β-cryptoxanthin, and β-carotene, are some of the most important pigments used in a wide range of industries [19]. Most of the health benefits of carotenoids in the human body are generally attributed to their antioxidant, anti-inflammatory, anti-cancer, and neuroprotective activities [20]. Additionally, carotenoids may have health-promoting effects mediated by other mechanisms, such as pro-oxidant action or modulation of immune functions [21].

Astaxanthin is an important secondary carotenoid with potent antioxidant properties. Therefore, it is widely used in numerous fields, including food production, chemical and pharmaceutical industries, as well as in aquaculture [22]. Astaxanthin can be obtained by chemical synthesis or biosynthesis. Chemical synthesis is complex, and the resulting product shows weak antioxidant activity. Biosynthesised astaxanthin, on the other hand, is much safer and has been adopted by the food and healthcare industries [23]. Microalgae are considered as the richest source of carotenoids. Carotenogenic algae accumulate β-carotene or ketocarotenoids as secondary metabolites, including astaxanthin, adonixanthin, and canthaxanthin. Astaxanthin is produced by various microalgae, including *Haematococcus lacustris*, *Acetabularia acetabulum*, *Chlainomonas rubra*, *Chromochloris, zofingiensis*, and *Diacronema vlkianum* [24]. Some microalgae, such as *Dunaliella salina* and *Trentepohlia*, primarily produce β-carotene. In *Euglena sanguinea*, astaxanthin and adonixanthin are the predominant carotenoids, while canthaxanthin is found in *Chlorosarcinopsis bastropiensis* and *Halochlorella rubescens* [25]. Recently, Nasri et al. showed that ZnO nanoparticles enhanced astaxanthin production in the chlorophyte *H. lacustris* and achieved maximum astaxanthin production at a ZnO nanoparticle concentration of 100 μg mL^−1^ [26]. Microalgae also produce other important carotenoids, such as β-carotene, lutein, β-cryptoxanthin, and canthaxanthin [27].

Astaxanthin also offers protection against tumour formation, increased stress, potentiation of B and T cell proliferation, decreased DNA fragmentation and immunity, and strengthened animals [28]. Appropriate amounts of astaxanthin enhance the antioxidant capacity and immunity of aquatic organisms, as well as improve their colour and growth rate [29]. Additionally, β-carotene is a natural carotenoid that is a precursor of vitamin A. Naturally derived β-carotene is highly biologically active and plays an important role in human health, showing therapeutic efficacy in several eye diseases and demonstrating antioxidant and anti-aging effects [30]. Lutein, a natural yellow carotenoid produced by plants and microorganisms, has strong antioxidant activity and has several beneficial effects on the human body. Lutein is mainly present in the cornea, where it contributes to healthy vision; therefore, several eye-protective lutein supplements are commercially available [31]. Fucoxanthin, the most abundant natural carotenoid, accounts for approximately 10% of the global carotenoid bioproduction and has been licensed as a nutraceutical [32]. Moreover, it is currently under consideration for regulatory approval by health and food safety authorities in different countries for its use in supplements and other products [33]. Zeaxanthin, a dihydroxy derivative of β-carotene, is yet another nutritional carotenoid found as a natural pigment in fruits and vegetables, as well as in the skin and eyes in the human body, where it plays an important role in protecting eyesight [34].

*Dysmorphococcus* is an algal genus within the family *Phacotaceae* of Chlorophyta. Under normal conditions, *Dysmorphococcus* cells form 4–8 or, occasionally, 16 dividing cells, which are 20–30 μm in diameter [35]. Zohir et al. isolated a strain of *D. globosus* in the Indian Himalayas and named it *D. globosus*-HI. Its cells had a 100–200 μm diameter, and each cell divided into 4–16 daughter cells. It may be a potential source of natural astaxanthin [36].

Enrichment of microalgae producing astaxanthin as a feed additive with Zn^2+^ can alleviate the symptoms of malnutrition in aquatic animals, simultaneously providing a mechanism for wastewater treatment. In addition, as candidate organisms for the synthesis of astaxanthin and Zn^2+^ accumulation, microalgae are crucial raw materials for functional foods and feeds used for consumption by fish and humans. Research on this issue is of paramount importance, as it may help in the struggle against hidden malnutrition of people around the world. Therefore, in the present study, we examined the effects of externally applied Zn^2+^ on the growth, physiology, chlorophyll fluorescence, intracellular Zn^2+^ content, and carotenoid content of the microalgal species *D. globosus*.

## 2. Results and Discussion

### 2.1. Morphological and Ultrastructural Observation and Identification

**Figure 1:**
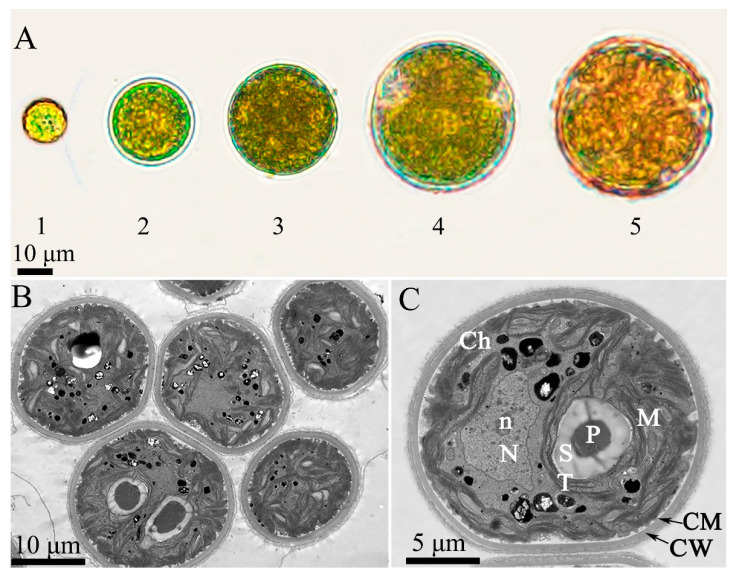
Morphological study of the new strain *Dysmorphococcus globosus* ZY24. (**A**) Morphological changes during growth of the microalga *D. globosus*. (**A1**) A motile cell under vegetative growth conditions (first day); (**A2**) initially immotile cell under vegetative growth conditions (second day); (**A3**) medium-term immotile cell under vegetative growth conditions (third day); (**A4**) mature cell under vegetative growth conditions (fifth day); (**A5**) immotile cell under inductive growth conditions (seventh day); (**B**,**C**) TEM images of ZY24. Ch: chloroplast; CM: cell membrane; CW: cell wall; M: mitochondria; N: nucleus; n: nucleolus; P: pyrenoid; S: starch; T: thylakoids.

**Figure 2:**
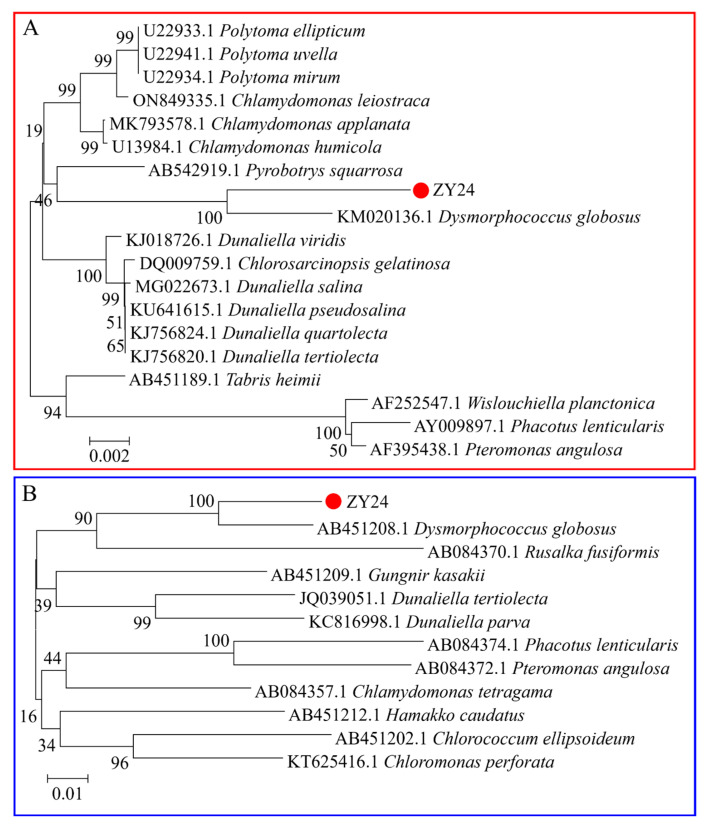
Phylogenetic trees of the isolated microalgal strain showing relatedness of its *18S* rRNA (**A**) and *psaB* (**B**) to previously obtained sequences of other algal species. The branch lengths are proportional to the evolutionary distances. Bootstrap values (>50%) from the bootstrap test (1000 replicates) are shown next to the phylogenetic trees. The scale bar shows the number of substitutions for each locus in the multiple comparison.

Morphological characteristics of the isolated microalgae were observed under a light microscope (IX73P2F, Olympus, Tokyo, Japan). The experimental strain was a unicellular dark-green microalga, with most cells in an aggregated state. The cells illustrated in Figure 1A were 10–40 µm in size. The diameter of the young green cells was approximately 10 mm, and the common cell size at maturity was approximately 40 mm. Under suitable culture conditions, microalgae had two flagella in their motile form in the green vegetative cell stage. When cells suffer from nutrient starvation or environmental stress factors, the colour of some of them changed from green to orange-red, which could indicate the accumulation of carotenoids. Further observations were performed using transmission electron microscopy (TEM; JEOL, JEM-1400-FLASH, Tokyo, Japan), and the main structures are shown in Figure 1B, C. An oblique section through a *D. globosus* ZY24 cell shows that one to two large pyrenoids were present, surrounded by a homogeneous starch sheet and thylakoids from both ends and on all sides. The cells had thick cell walls and were surrounded by a cell membrane. Chloroplasts occupied most of the cell volume. The mitochondria were present in the cytoplasm. The oblique section also showed the nucleus and nucleolus. Neighbour-joining tree clustering clearly showed the genetic relationships of strain ZY24 within the genus *Dysmorphococcus* (Figure 2). The *18S rRNA* and *psaB* sequences showed similarity of the newly isolated strain with the sequenced strain of *D. globosus* (SAG 20-1) according to reference sequences of *18S rRNA* (KM020136.1) and *psaB* (AB451208.1). The NCBI accession numbers for this algal strain’s gene sequences of *18S rRNA* and *psaB* are PQ002471 and PQ046780, respectively. The structure of *D. globosus* cells observed under the microscope was similar to that of *D. globosus*-HI cells [36]. Our algal strain was named *D. globosus* ZY24.

### 2.2. Comparison of Zn-Enriched Microalgal Cultures

#### 2.2.1. Effects of Zn Stress on Strain ZY24 Grown in Darkness

The characteristics of microalgal *D. globosus* strain ZY24 grown in media with different Zn^2+^ concentrations were investigated during its growth in darkness. The phenotype of Zn-stressed microalgae cultivated in darkness showed no significant changes (Figure S1). Further, the density of microalgal cells grown under all tested Zn^2+^ concentrations increased on the first day of treatment; however, after day 1, all treatment groups showed significant growth inhibition (Figure S2). These results indicated that Zn^2+^ concentrations higher than 22.75 mg L^−1^ limited microalgae growth.

#### 2.2.2. Chlorophyll Fluorescence of Strain ZY24 Under High Zn^2+^-Induced Stress in the Dark

The *F*v/*F*m parameter, which can be used to assess the degree of photoinhibition experienced by a plant, showed only slightly variation under non-stress conditions regardless of species or growth conditions; however, it decreased significantly under stress [37]. Photosynthesis is highly sensitive to the presence of metals, and Zn^2+^ has a strong effect on plant photosynthetic activity. In general, *F*v/*F*m and *Y*(II) represent the potential and actual quantum efficiency of photosystem II (PSII), respectively, and *F*v/*F*m can directly reflect the degree of PSII damage in the cells. Thus, a significant decrease in *F*v/*F*m indicates that the algae are under stress [38]. In this study, *F*v/*F*m and *Y*(II) values decreased significantly with an increase in Zn^2+^ concentration in the medium (Figure S3A–C). On the seventh day of treatment, the value of *F*v/*F*m in the 364 mg L^−1^ Zn^2+^ concentration treatment group was reduced to 0.21, which was 1.8 times lower than that of the control group, and the value of *Y*(II) was reduced to 0.1, which was 2.9 times lower than that of the control group. These experimental data suggest that high concentrations of Zn^2+^ severely affect the photosynthetic efficiency of ZY24.

#### 2.2.3. Zn Accumulation in *Dysmorphococcus globosus* Strain ZY24 Grown in the Dark

Several microalgal species effectively adsorb heavy metals both in mono-metallic and poly-metallic systems, which is why they are often used as bioremediation agents [39]. In our study, algae from treatment groups grown in the presence of 0.0, 45.5, and 364.0 mg Zn L^−1^ were selected for determination of the cellular Zn content. On day 7 after treatment initiation, the Zn content was 22,453 mg kg^−1^ in ZY24 cultures grown in the presence of 364 mg Zn^2+^ L^−1^ (Figure S4).

### 2.3. Zn Endurance Test of Strain ZY24 Under White Light Conditions

#### 2.3.1. Morphological Changes in Zn^2+^-Treated Cells

**Figure 3:**
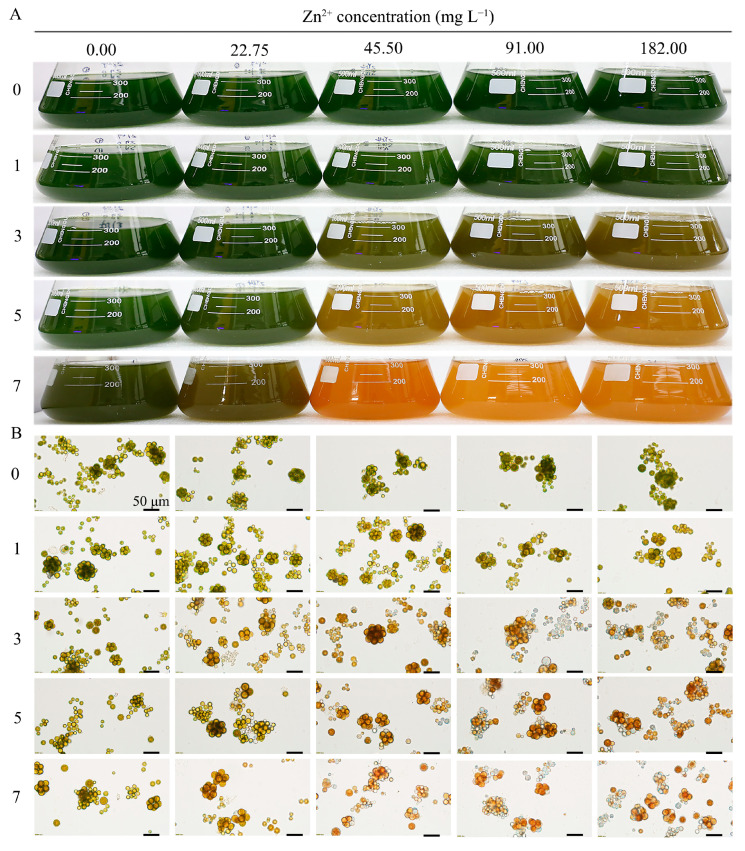
The phenotype of Zn^2+^-stressed *Dysmorphococcus globosus* strain ZY24 during 7 days of treatment with different concentrations of Zn^2+^. (**A**) Colour change in ZY24 culture medium after treatment with different Zn^2+^ concentrations. (**B**) Effects of treatment with different Zn^2+^ concentrations on ZY24 cells. The scale in all cell maps is 50 μm.

As shown in Figure 3A, there was no significant change in algae treated with 0 mg L^−1^ Zn^2+^ over 7 days. However, at Zn^2+^ concentrations higher than 22.75 mg L^−1^, the microalgae changed their colour from green to brown, indicating that they were affected by high Zn^2+^ ion concentrations. On the seventh day of treatment, microalgae exposed to 45.5 mg L^−1^ Zn^2+^ turned orange-red. The cells were observed under a light microscope (Figure 3B), and on the third day of treatment, at concentrations above 22.75 mg L^−1^ Zn^2+^ ions, the colour of the cells changed to orange-yellow, likely owing to pigment accumulation. By day 7 of treatment, the most significant colour change in the microalgae was observed in the 45.5 mg L^−1^ treatment group.

#### 2.3.2. Effects of Zn Stress on Microalgal Growth

**Figure 4:**
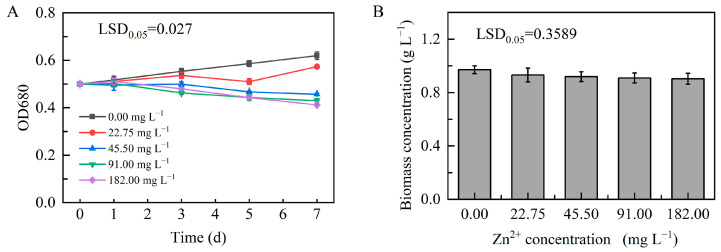
Effect of Zn^2+^-induced stress on the growth of microalgal *Dysmorphococcus globosus* strain ZY24 under white light conditions. (**A**) Changes in optical density at 680 nm from days 0 to 7 of treatment; (**B**) strain ZY24 biomass accumulation after 7 days of treatment. Each data point represents the mean of three replicates; error bars indicate the standard error of the mean. The statistical significance of the difference between the means was analysed using Duncan’s Least Significant Difference (LSD) test.

At the optimal concentration, Zn promoted normal microalgal growth. For example, Liu et al. investigated the combined effects of Zn^2+^ and estrone on the microalga *Chlorella sorokiniana*. They showed that low concentrations of Zn^2+^ in the culture medium promoted its growth and photosynthetic activity, whereas high concentrations inhibited these processes [40]. Similarly, in this study, we investigated the growth characteristics of microalgal *D. globosus* strain ZY24 in culture media containing different Zn^2+^ concentrations (Figure 4). Microalgal cell density in all treatment groups increased on day 1 of treatment. However, significant growth retardation was observed in all treatment groups starting on day 3 of treatment, except in the 0.00 and 22.75 mg L^−1^ groups. Later, on day 7 of treatment, optical cell density at 680 nm was only 0.412, and biomass was 0.903 g L^−1^ in the 179.36 mg L^−1^ treatment, indicating an apparent growth reduction compared to the control group. These results showed that high Zn^2+^ concentrations strongly limited microalgal cell growth.

#### 2.3.3. Chlorophyll Fluorescence Analysis

**Figure 5:**
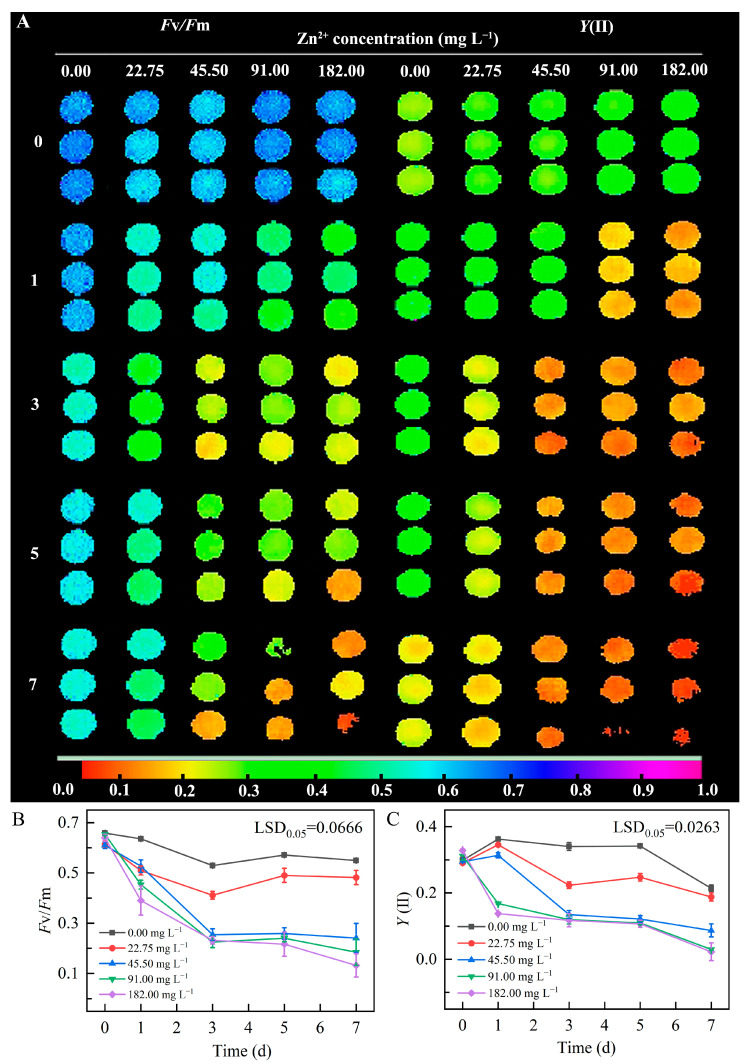
Changes in chlorophyll fluorescence parameters in microalgal *Dysmorphococcus globosus* strain ZY24 cultivated in the presence of different concentrations of Zn^2+^ over 7 days. (**A**–**C**) show the phenotype, maximum quantum efficiency, and actual quantum yield of PS II photochemistry, respectively. Each data point represents the meaning of three replicates; error bars indicate the standard error of the mean. The statistical significance of the difference between the means was analysed using Duncan’s Least Significant Difference (LSD) test. *F*v/*F*m is associated with the maximum photochemical quantum yield of PSII; *Y*(II) is an important parameter for measuring the efficiency of converting light energy to chemical energy in plant photosynthesis.

In plants, especially in algae, metal toxicity usually first affects PSII. Therefore, we assessed the effect of different concentrations of Zn^2+^ on microalgae by determining chlorophyll fluorescence parameters, such as the maximum quantum efficiency of PSII (*F*v/*F*m) and quantum efficiency of PSII [*Y*(II)] [41]. *F*v/*F*m is associated with the maximum photochemical quantum yield of PSII, where *F*v represents the maximum variable fluorescence (*F*m − *F*o), and *F*m is the maximum chlorophyll (chl) *a* fluorescence yield in the dark-adapted state [42]. Specifically, the data showed that *F*v/*F*m followed an overall decreasing trend, and when Zn^2+^ concentration exceeded 45.5 mg L^−1^, this reduction became significant, indicating that high Zn^2+^ concentrations significantly suppressed microalgal photosynthesis (Figure 5A–C) over the first 5 days of treatment. Further, 7 days after treatment initiation, the most significant level of stress was caused by 182 mg L^−1^ Zn^2+^ in the growth medium. This finding was consistent with the previously mentioned maximal reduction of cellular absorbance in the same treatment group (Figure S4).

#### 2.3.4. Chlorophyll Content

**Figure 6:**
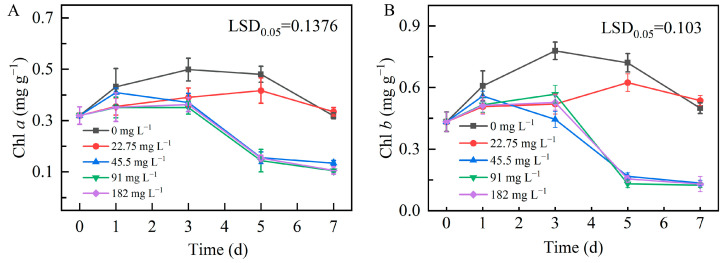
Chlorophyll contents in microalgal *Dysmorphococcus globosus* strain ZY24 cultivated in Zn^2+^-enriched media. (**A**) Chlorophyll *a*. (**B**) Chlorophyll *b*. Each data point represents the mean of three replicates. Error bars indicate the standard error of the mean. The statistical significance of the difference between the means was analysed using Duncan’s Least Significant Difference (LSD) test.

Chlorophyll is one of the most critical pigments in the majority of photosynthesising organisms because it allows algae and plants to absorb the electromagnetic energy of light and convert it into chemical energy stored in covalent bonds [43]. The chlorophyll content in microalgal cells varies depending on the environmental factors. The effects of Zn^2+^ on chlorophyll *a* and *b* contents in ZY24 microalgae are shown in Figure 6. The contents of both types of chlorophyll decreased in a concentration-dependent manner when microalgae were cultivated in Zn^2+^-supplemented media. Thus, the chlorophyll content was significantly reduced at Zn^2^⁺ concentrations higher than 45.5 mg L^−1^ beginning on day 3 after treatment initiation, presumably owing to the cytotoxic effects of high Zn^2+^ levels.

#### 2.3.5. Pigment Content

**Figure 7:**
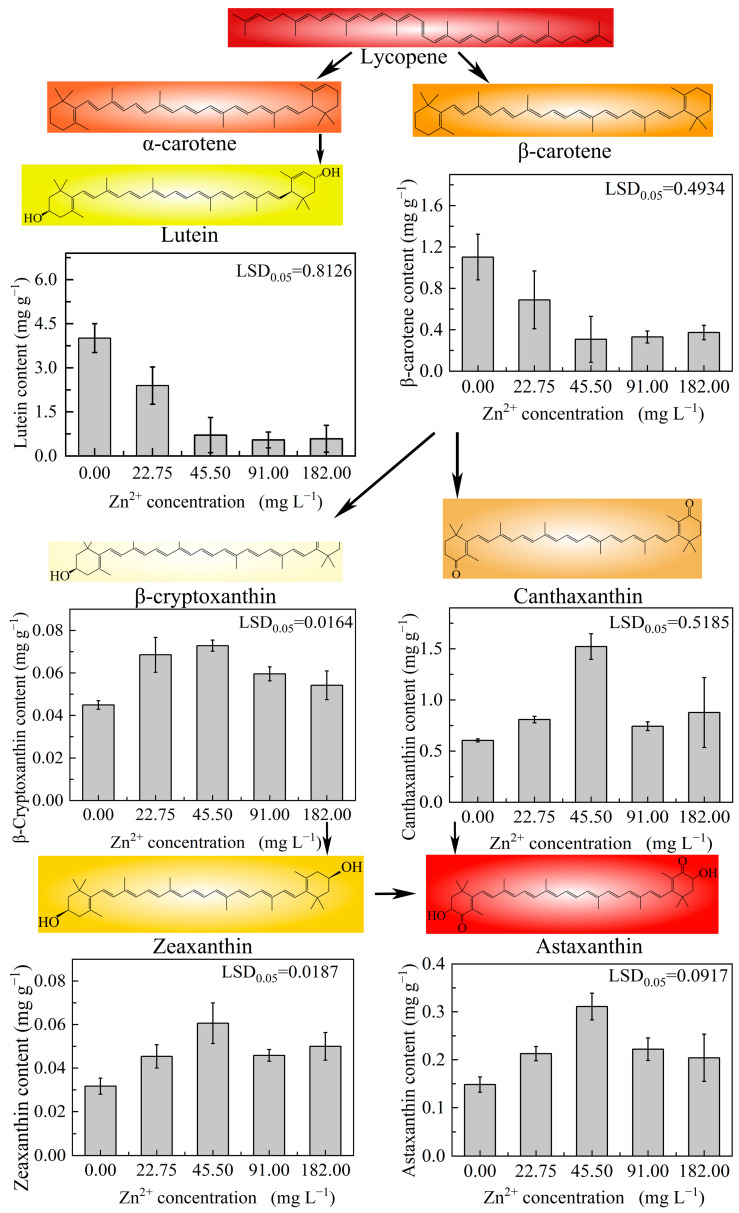
Contents of β-cryptoxanthin, lutein, β-carotene, astaxanthin, zeaxanthin, and canthaxanthin in *Dysmorphococcus globosus* strain ZY24 determined on day 7 of treatment. Each data point represents the mean of three replicates; error bars indicate the standard error of the mean. The statistical significance of the difference between the means was analysed using Duncan’s Least Significant Difference (LSD) test.

Other microalgae, such as *Chlorella vulgaris* and *D. salina*, are potential additional sources of carotenoids. Further, several species of microalgae, such as *D. salina*, *A. platensis*, and some diatoms, are good sources of β-carotene, lutein, canthaxanthin, and astaxanthin [44]. In this study, high Zn^2+^-induced stress promoted pigment formation in strain ZY24, such that astaxanthin, zeaxanthin, β-cryptoxanthin, and canthaxanthin contents significantly increased in microalgae cultivated in the presence of 45.5 mg L^−1^ Zn^2+^, reaching values of 0.311, 0.061, 0.073, and 1.522 mg g^−1^, respectively (Figure 7). However, contents of all these pigments began to decrease at Zn^2+^ concentrations greater than 91 mg L^−1^. Indeed, excessively high Zn^2+^ concentrations reduced pigment production and ultimately caused cell death. In particular, β-carotene and lutein contents were inversely proportional to Zn^2+^ concentrations, likely because Zn^2+^ stress caused inhibition of pigment synthesis concomitant with the conversion of existing β-carotene to canthaxanthin and zeaxanthin, and ultimately, of zeaxanthin to astaxanthin [45]. Chekanov et al. showed that astaxanthin and β-carotene were the predominant pigments in the cells of the new microalgal strain *Bracteacoccus aggregatus*, BM5/15, comprising 48% and 13%, respectively [46]. In contrast, the main pigments in *D. globosus* strain ZY24 were canthaxanthin, astaxanthin, and β-carotene, present in ratios of 51%, 10%, and 10%, respectively. The fractions of astaxanthin and β-carotene were high in these algal species. Biotechnological approaches may be employed to enhance their astaxanthin content.

#### 2.3.6. Zn Accumulation in Microalgae

**Figure 8:**
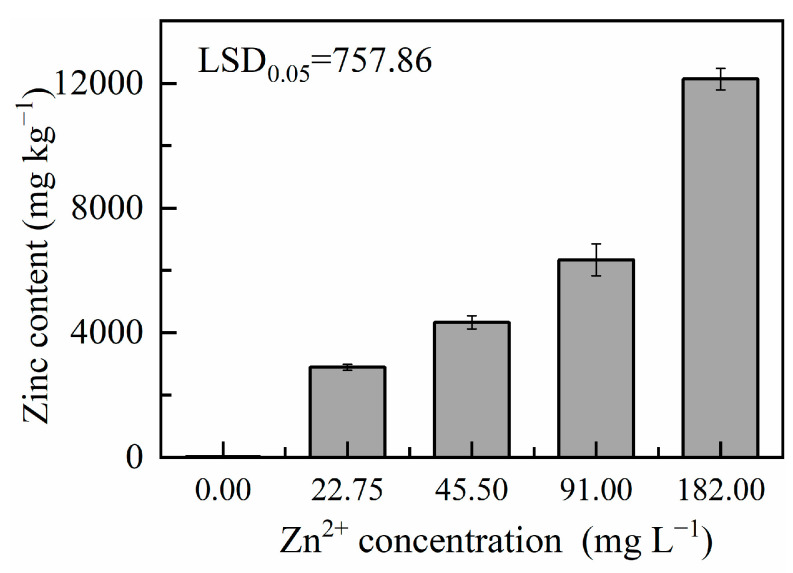
Zn^2+^ accumulation in *Dysmorphococcus globosus* strain ZY24 harvested at various initial Zn^2+^ concentrations in the growth medium. Each data point represents the mean of three replicates; error bars indicate the standard error of the mean. The statistical significance of the difference between the means was analysed using Duncan’s Least Significant Difference (LSD) test.

Microalgae are characterised by a high light-energy transformation efficiency, fast growth rate, and high capacity to accumulate specific organic mineral elements. They can be used as biological carriers of Zn^2+^, which could improve their health-promoting properties. In addition, they play a role in bio-enrichment and the transformation of mineral elements [47,48]. Higher levels of Zn^2+^ in microalgal cells due to increasing Zn^2+^ concentrations in the culture medium indicated that *D. globosus* strain ZY24 can accumulate Zn^2+^ (Figure 8). Indeed, astaxanthin concentrations reached approximately 0.311 mg g^−1,^ and total Zn levels in microalgae reached 4337 mg kg^−1^ at a 45.5 mg L^−1^ Zn^2+^ concentration. This result proves that *D. globosus* strain ZY24 accumulates Zn^2+^ ions and produces astaxanthin at the same time, which is of great significance for solving the problem of hidden starvation in people and the remediation of environmental toxicity associated with the presence of heavy metals [49]. Carotenoid accumulation in *D. globosus* strain ZY24 in the presence of high levels of Zn in the growth medium can be considered a beneficial feature. As heavy metals such as Zn induce reactive oxygen species production, carotenoids function as antioxidants, i.e., they reduce reactive oxygen species levels and protect cellular metabolism [50]. In addition, carotenoids may be involved in metal detoxification and metabolic regulation under stress, as recent studies on the physiological functions of carotenoids under heavy metal stress showed [50]. These adaptive mechanisms allow *D. globosus* strain ZY24 to maintain the structure and function of the mother cells in the context of Zn-induced oxidative stress. Interestingly, Price et al. studied the effects of Zn^2+^ on *C*. *vulgaris* at different pH levels and showed that the accumulation of this cation decreased as pH increased [51]. Therefore, changes in environmental factors may also affect the Zn^2+^ adsorption capacity by microalgae, an issue that needs further in-depth study.

## 3. Materials and Methods

### 3.1. Sample Collection, Isolation, and Growth Conditions

Microalgal *Dysmorphococcus globosus* (CCTCC NO: M 2024627) strain No. ZY24 was isolated in Ziyang County, Ankang City (32°49′31″ N, 108°60′92″ E). The collected microalgal samples were cultivated in the BG11 medium for growth. Purified cultures were obtained by successive streaking of the samples on BG11 solid medium containing antibiotics (50 μg mL^−1^ ampicillin and 100 μg mL^−1^ cefotaxime) under sterile conditions. Purified microalgal cultures were incubated in liquid medium at 25 ± 1 °C under white light (30 μmol m^−2^ s^−1^) and a 12:12 h light–dark regime, with continuous shaking at 150 rpm. Microalgal samples (0.6 mL) were placed in a 1.5 mL centrifuge tube with a cell density of 6 × 10^5^ cells/mL; then, 0.6 mL of 30% glycerol was added to each sample prior to inverting, thoroughly mixing, and freezing at −80 °C. The BG11 medium composition was as follows: NaNO_3_ 1.5 g, K_2_HPO_4_ 0.04 g, MgSO_4_∙7H_2_O 0.075 g, CaCl_2_∙2H_2_O 0.036 g, Na_2_CO_3_ 0.02 g, citric acid 0.006 g, ferric ammonium citrate 0.006 g, H_3_BO_4_ 0.00286 g, MnCl_2_∙H_2_O 0.00181 g, ZnSO_4_∙7H_2_O 0.000222 g, Na_2_MoO_4_ 0.00039 g, CuSO_4_∙5H_2_O 0.000079 g, Co(NO_3_)_2_∙6H_2_O 0.000049 g, pH 7.0 at 25 °C [52].

### 3.2. Morphological Characterisation Using Light Microscopy and TEM

A light microscope (IX73P2F, Olympus, Japan) was used to observe the isolated microalgal strains. The microscope was connected to a computer for real-time imaging of the cells using the CellSens Dimension 3.2 software (Olympus, CellSensDimension, Japan). The microscope was adjusted to an appropriate magnification, and the software displayed the moving state and structural characteristics of *D. globosus* strain ZY24. For TEM (JEOL, JEM-1400-FLASH, Japan), cell cultures were centrifuged, and the resulting pellet was fixed for 2 h at 4 °C in 2.5% using glutaraldehyde (*v*/*v*) diluted in a 0.2 M sodium cacodylate buffer (pH 7.4) containing 10% or 20% NaCl. Following a buffer wash, the samples were post-fixed with 2% osmium tetroxide buffered with sodium cacodylate, dehydrated through increasing concentrations of ethanol, and embedded in epoxy resin (Fluka, Buchs, Switzerland). Ultrathin sections were stained for 20 min with 3% uranyl acetate, washed, and then stained for 10 min [53] with lead citrate before observation using a transmission electron microscope.

### 3.3. Molecular Identification and Phylogenetic Analysis

Microalgal isolates (10 mL) were centrifuged at 10,000× *g* rpm and 25 °C for 30 min, and DNA was extracted using the DNAsecure Plant Kit (TIANGEN, Beijing, China). Each extracted DNA sample was processed by electrophoresis (Bio-Rad, 164–5050, Bio-Rad Laboratories, Hercules, CA, USA) using a UVP Benchtop Transilluminator (Upland, CA, USA). PCR was performed using Q5 High Fidelity DNA Polymerase (New England Biolabs, Ipswich, MA, USA). The obtained DNA samples were amplified by PCR using specific primers *(18S ribosomal RNA* gene, *18S rRNA*-F: 5′-AACCTGGTTGATCCTGCCAGT-3′, *18S rRNA*-R: 5′-TGATCCTTCTGCAGGTTCACCTAC-3′; photosystem I P700 chlorophyll *a* apoprotein A2, *psaB*-F: 5′-GGTGGTTTTCATCCACAAACTC-3′, *psaB*-R: 5′-GAACCACGTGCATCTAAAGCACCT-3′) [54]. These primers targeted different regions of the microalgal genome for comprehensive analysis. PCR was performed with initiation at 98 °C for 30 s, followed by 35 cycles of denaturation at 95 °C for 30 s, annealing at 55 °C for 30 s, extension at 72 °C for 1 min, and termination at 72 °C for 1 min. The PCR products were run in a 1.5% agarose gel at 200 V for 25 min, and the DNA was subsequently electrophoresed and used for direct sequencing based on the Sanger’s dideoxy method [55]. Homologous sequences were searched using BLAST (Basic Local Alignment Search Tool; http://blast.ncbi.nlm.nih.gov/ (accessed on 27 June 2024)) against the NCBI GenBank database to identify similar sequences for alignment and analysis [56]. With the help of the MEGA 5.05 software, the sequences were aligned by ClustalW, and the phylogenetic tree was reconstructed from nucleotide sequence alignments using the neighbour-joining method (bootstrap method; 1000 bootstrap replicates) [57,58,59].

### 3.4. Zn^2+^ Tolerance Test

#### 3.4.1. Cultivation Methods

Microalgae were cultivated to the late exponential stage and then set aside. Subsequently, they were transferred to fresh BG11 medium, and the initial microalgal concentration was adjusted to an optical density (OD) of 0.5 at 680 nm using a spectrophotometer (Metash, UV-9000, Shanghai, China) before dispensing into 50 mL conical flasks for batch testing. Then, ZnSO_4_∙7H_2_O was added to the sterile medium to achieve final Zn^2+^ concentrations of 0.00, 22.75, 45.50, 91.00, 182.00, and 364.00 mg L^−1^, and the flasks were incubated for 7 days at 25 ± 1 °C under constant darkness and continuous shaking at 150 rpm.

#### 3.4.2. Microalgal Growth Curve Determination

OD of the microalgal strain ZY24 was measured at 680 nm using a spectrophotometer at 0, 1, 3, 5, and 7 days of incubation by aspirating 200 μL of the fresh microalgal solution, and a growth curve was plotted. To measure algal biomass, algal solutions were centrifuged and washed three times with distilled water. The resulting cell pellets were lyophilised for 36 h and then weighed.

#### 3.4.3. Chlorophyll Fluorescence Measurements

Chlorophyll fluorescence was determined using Maxi IMAGING-PAM (WALZ, Effeltrich, Germany). Samples were thoroughly mixed and 200 μL of the suspension was pipetted into the wells of black 96-well plates. Measurements were repeated three times for each sample. The values of *F*v/*F*m and *Y*(II) were obtained using Maxi IMAGING-PAM (WALZ, Effeltrich, Germany). In the ImagingWin software ver. 2.56, the intensity of photosynthetically active radiation (PAR) was set to 0 and 600 μmol m^2^ s^−1^, with an irradiation time of 10 s for each intensity. *F*t was maintained at 0.1 to ensure experimental accuracy. The samples were dark-adapted for 5 min before *F*v/*F*m measurements. At this point, PAR was set to 0 μmol m^2^ s^−1^, and *F*v/*F*m was measured by clicking the (*F*o, *F*m) button in the software’s operation window after 1 s. Following this, PAR was adjusted to 600 μmol m^2^ s^−1^, photochemical light (AL) was activated, and measurements were completed by clicking the SAT-Pulse button after 5 min. Each measurement was repeated three times for every sample.

#### 3.4.4. Zn Accumulation in Microalgae

Microalgal cells were lyophilised using a freeze dryer (Scientz, SCIENTZ-10ND, Ningbo, China) and analysed using inductively coupled plasma mass spectrometry (ICP-MS) (7800, Agilent Technologies, Santa Clara, CA, USA). Solid samples weighing 0.1 g (accurate to 0.001 g) were placed in a microwave digestion inner jar. Then, 5 mL of nitric acid was added, the lid was closed tightly, and the sample was digested for 1 h or overnight according to the standard operation procedure of the microwave digestion apparatus. After cooling, the lid was slowly opened and rinsed with a small amount of water. Then, the tank was placed in an ultrasonic water bath, degassed for 2–5 min under ultrasonication, and samples were diluted with water to 25 or 50 mL and analysed using ICP-MS. Parameters of ICP-MS were as follows: radio frequency power of 1500 W, plasma gas flow rate of 15 L min^−1^, carrier gas flow rate of 0.8 L min^−1^, auxiliary gas flow rate of 0.8 L min^−1^, and helium gas flow rate of 4 mL min^−1^. The chamber temperature stabilised to the first decimal point with an atomisation of 2 °C. The lifting rate for the sample was found to be at 0.3 r s^−1^. This was performed over three distinct time periods on the same sample to determine the inter-session reliability of the method.

### 3.5. Synergistic Effects of Zinc and Astaxanthin

#### 3.5.1. Culturing Method

Microalgae were cultivated to the late exponential stage and then set aside. Subsequently, they were transferred into fresh BG11 medium. The medium was replaced, the initial microalgal concentration was adjusted to an OD of 0.5 at 680 nm using a spectrophotometer, and the cells were dispensed into 300 mL conical flasks for batch testing. ZnSO_4_∙7H_2_O was added to a sterile medium to achieve Zn^2+^ concentrations of 0.00, 22.75, 45.50, 91.00, and 182.00 mg L^−1^ under the same temperature conditions as mentioned above; then, cultures were incubated for 7 days under a light intensity of 600 μmol m^−2^ s^−1^ and a photoperiod of 24 h. All experiments were replicated three times.

#### 3.5.2. Measurement of Chlorophylls *a* and *b*

The levels of chlorophyll *a* and *b* were determined by reference to Lightenthaler’s (1987) method with some modifications [60]. First, 2 mL of fresh microalgal suspension was transferred to a centrifuge tube and centrifuged at 5000× *g* rpm for 5 min. Subsequently, the supernatant was removed and approximately 1 mL of the initial suspension was retained. Then, the microalgal suspension was transferred to a pre-weighed 2 mL centrifuge tube, and deionised water was added to bring the solution to 1 or 1.5 mL. The tube was then centrifuged at 6000× *g* rpm for 5 min, the supernatant was removed (as much water as possible was removed in this step), and the weight of the centrifuge tube was measured (the difference in the weight of the tube between the two centrifugations was considered the wet weight of the microalgal solution). Thereafter, precautions were taken to avoid any exposure to light. Two small steel beads were added to the 2 mL centrifuge tube, and microalgae were ground using a tissue grinder. Subsequently, 1.5 mL of methanol was added to the tube before centrifuging at 6000× *g* rpm for 3 min to remove the residue from the tube walls. The residue was again centrifuged at 6000× *g* rpm for 3 min and then crushed using an ultrasonic crusher. After ultrasonication, the sample was left in the dark at room temperature for 2 h. After centrifugation, the residue was allowed to reach the bottom, and 200 μL of the microalgal residue was pipetted to determine the absorbance at 652.4 and 665.2 nm using a spectrophotometer. Equations (1) and (2) were used to calculate chlorophyll *a* and *b* concentrations, expressed as mg L^−1^. Equation (3) was used to calculate chlorophyll *a* and *b* contents, expressed as mg g^−1^ [61].

Chl *a* = 16.72A_665.2_ − 9.16A_652.4_ (1)

Chl *b* = 34.09A_652.4_ − 15.28A_665.2_ (2)

(3) $$Y=(C\times \frac{V}{W\times \left(1-wc\right)\times 1000})$$

where Chl *a* and Chl *b* are the concentrations of chlorophyll *a* and chlorophyll *b* (mg L^−1^), respectively; A_665.2_ and A_652.4_ are the absorbance values at 665.2 and 652.4 nm, respectively; *Y* is the corresponding chlorophyll yield (mg. g^−1^ dry weight algae); *C* is the concentration (mg L^−1^) of chlorophyll *a* or chlorophyll *b*; and *V*, *W*, and *wc* are the volume of the extract sample (mL), the wet weight of algae (g), and the mass fraction of water in the fresh algae, respectively.

#### 3.5.3. High-Performance Liquid Chromatography (HPLC) Analysis of Astaxanthin and Other Carotenoids

A freeze-dried microalgal sample (10 mg) was accurately weighed in a dry glass homogeniser, and 0.2 mL of a dichloromethane–methanol solution was added, followed by complete grinding to break the cell walls. The suspension was then transferred to a 10 mL centrifuge tube, the glass homogeniser was washed three times with 2 mL of dichloromethane–methanol solution, and the extracts were combined. The resulting suspension underwent ultrasonic treatment for 5 min, followed by centrifugation at 8000× *g* rpm and 5 °C for 5 min. The supernatant was then transferred to a 10 mL centrifuge tube and 2 mL of dichloromethane–methanol solution was added. These steps were repeated more than three times, until the extracted microalgal scum was white, indicating that the supernatants were combined. The dichloromethane–methanol solution was fixed at 6 mL and left to stand for 15 min. Then, 5 mL of the supernatant was accurately pipetted into another 10 mL centrifuge tube; 0.7 mL of sodium hydroxide–methanol solution was added and the mixture was vortexed and then sealed and reacted in a fridge at 5 °C overnight for 12–14 h. Subsequently, 0.4 mL of a 2% phosphoric acid–methanol solution was added to the reaction mixture to neutralise the remaining alkali, mixed, and filtered through a 0.45 μm filter membrane. The filtrate was removed and used as the specimen solution. The intracellular pigment content was detected using HPLC on a 120EC-C18 column (150 mm × 3 mm, 2.7 μm) under the following conditions: mobile phase: solution A: 50% ultrapure water and 50% methanol containing 0.1% (*v*/*v*) formic acid, and solution B: 80% methyl tert-butyl ether and 20% methanol solution containing 0.1% (*v*/*v*) formic acid. The column temperature was set at 30 °C; the detection wavelength at 474 nm; the injection volume at 10 μL; and the flow rate at 0.2 mL min^−1^.

### 3.6. Statistical Analysis

Data are shown as the mean ± standard error of the mean (*n* = 3). The statistical significance of the difference between the means was analysed using Duncan’s Least Significant Difference (LSD) test. LSD_0.05_ is an essential concept in statistics for comparing the means of multiple groups and usually used when making various comparisons after an analysis of variance (ANOVA). If the difference between the means of the two groups is more significant than the LSD_0.05_ value, it can be assumed that the two groups are statistically different. A significant difference was compared based on the calculation of LSD_0.05_.

## 4. Conclusions

Zn^2+^-enriched microalgae represent a promising alternative as a fish food source, providing essential nutrients while at the same time lowering fish farming costs. Aquaculture practices incorporating Zn^2+^ into microalgal biomass can improve the nutritional composition of fish diets, resulting in better fish welfare, growth, and quality. Furthermore, cultivating Zn^2+^-enriched microalgae is a cost-effective and sustainable approach that takes advantage of the rapid growth and high biomass yield of microalgae to meet the nutritional needs of fish in the aquaculture industry. In this study, Zn^2+^ significantly changed the growth of *D. globosus* strain ZY24, with high Zn^2+^ concentrations strongly inhibiting growth, whereas low concentrations showed no significant effect on microalgae growth. Additionally, fluorescence activity measurements indicated that microalgal *D. globosus *strain ZY24 was tolerant to Zn^2+^ during long-term culture. Chlorophyll *a* and *b* contents reached their maximum values on day 3 of culture in high external Zn^2+^ concentrations. Thus, at 45.5 mg L^−1^, Zn^2+^ significantly increased the astaxanthin content in microalgal cells. Indeed, Zn^2+^ accumulation in microalgal cells increased proportionally with increasing external Zn^2+^ concentrations. The microalgal culture effectively increased Zn^2+^ contents and astaxanthin production at an external Zn^2+^ concentration of 45.5 mg L^−1^. Extensive molecular and physiological experiments on *D. globosus*, followed by thorough testing of the results in fish, will enable the development of a new and efficient fish diet. By evaluating the effects on fish growth and health and optimising the nutritional content, these studies will pave the way to creative and long-lasting aquaculture feed solutions. However, this study had limitations. Firstly, in the absence of reference genomic data for *D. globosus*, the astaxanthin synthesis pathway genes remain unclear. Secondly, the mechanisms responsible for Zn^2+^ enrichment and excretion in *D. globosus* are unclear. Therefore, further studies are needed to reveal the mechanisms of Zn^2+^ uptake and astaxanthin synthesis in *D. globosus* concerning the relevant genes and to develop approaches enhancing the astaxanthin content in this species through genetic modification.

## Data Availability

The data presented in this study are available from the corresponding author on request.

## Supplementary Materials

The following supporting information can be downloaded at: https://www.mdpi.com/article/10.3390/plants13233338/s1, Figure S1: Phenotype of *Dysmorphococcus globosus* strain ZY24 grown for 7 days in the dark for 24 h. Figure S2: Effect of Zn^2+^-induced stress on the growth of *Dysmorphococcus globosus* strain ZY24 under dark conditions. Figure S3: Changes in chlorophyll fluorescence of microalgae *Dysmorphococcus globosus* strain ZY24 cultivated under dark conditions in the presence of different Zn^2+^ concentrations over 7 days. Figure S4: Zinc accumulation in *Dysmorphococcus globosus* strain ZY24 harvested after 7 days of treatment with different initial Zn^2+^ concentrations under dark conditions. Figure S5: Liquid chromatograms of 0.00, 22.75, 45.50, 91.00, and 182.00 mg L^−1^ treatment groups on day 7, respectively.

## Abbreviations

Chl, chlorophyll; HPLC, high-performance liquid chromatography; ICP-MS, inductively coupled plasma mass spectrometry; OD, optical density; PSII, photosystem II; TEM, transmission electron microscopy.
