# Supplementary material for: Effects of Culture Medium Enrichment with Zinc on Astaxanthin Accumulation in a New Strain of the Microalga Dysmorphococcus globosus
Source: Plants (Basel). 2024 Nov 28;13(23):3338. doi: 10.3390/plants13233338 (PMC11644740; doi:10.3390/plants13233338)

Figure S1. Penotype of *Dysmorphococcus globosus* strain ZY24 grown for 7 days in the dark for 24 h. The phenotype of microalgae did not change significantly.

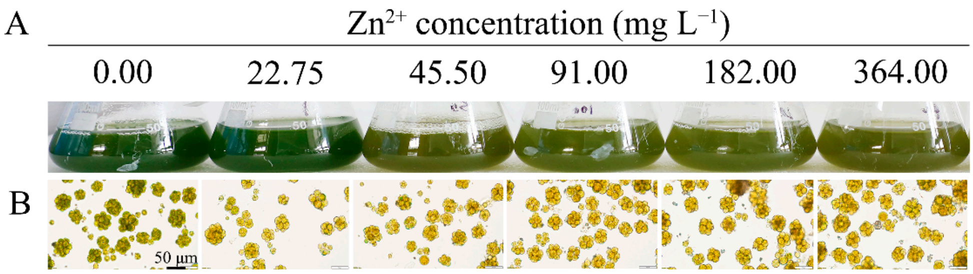

Figure S2. Effect of Zn^{2+}-induced stress on the growth of *Dysmorphococcus globosus* strain ZY24 under dark conditions. Except 0 mg L^{-1} Zn^{2+} concentration, all groups showed a decreasing growth trend. Each data point represents the mean of 3 replicates; error bars represent the standard error of the mean.

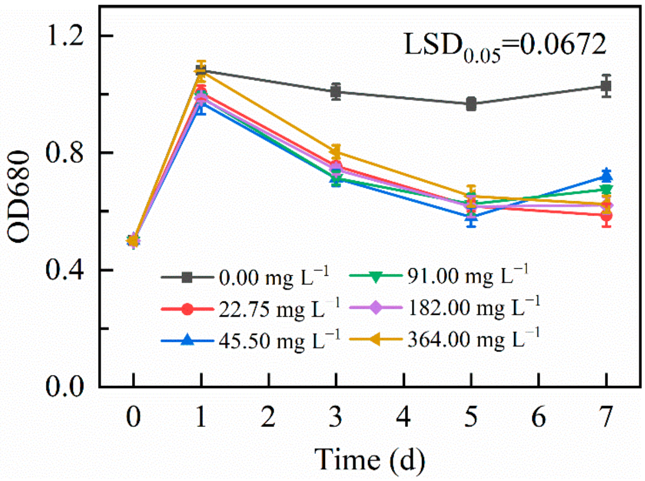

Figure S3. Changes in chlorophyll fluorescence of microalgae *Dysmorphococcus globosus* strain ZY24 cultivated under dark conditions in the presence of different Zn^{2+} concentrations over 7 days. Zn-induced variation of chlorophyll fluorescence parameters. A, B, and C show the phenotype and maximum quantum efficiency and actual quantum yield of PSII photochemistry, respectively. Each data point represents the mean of three replicates; error bars represent standard error of the mean.

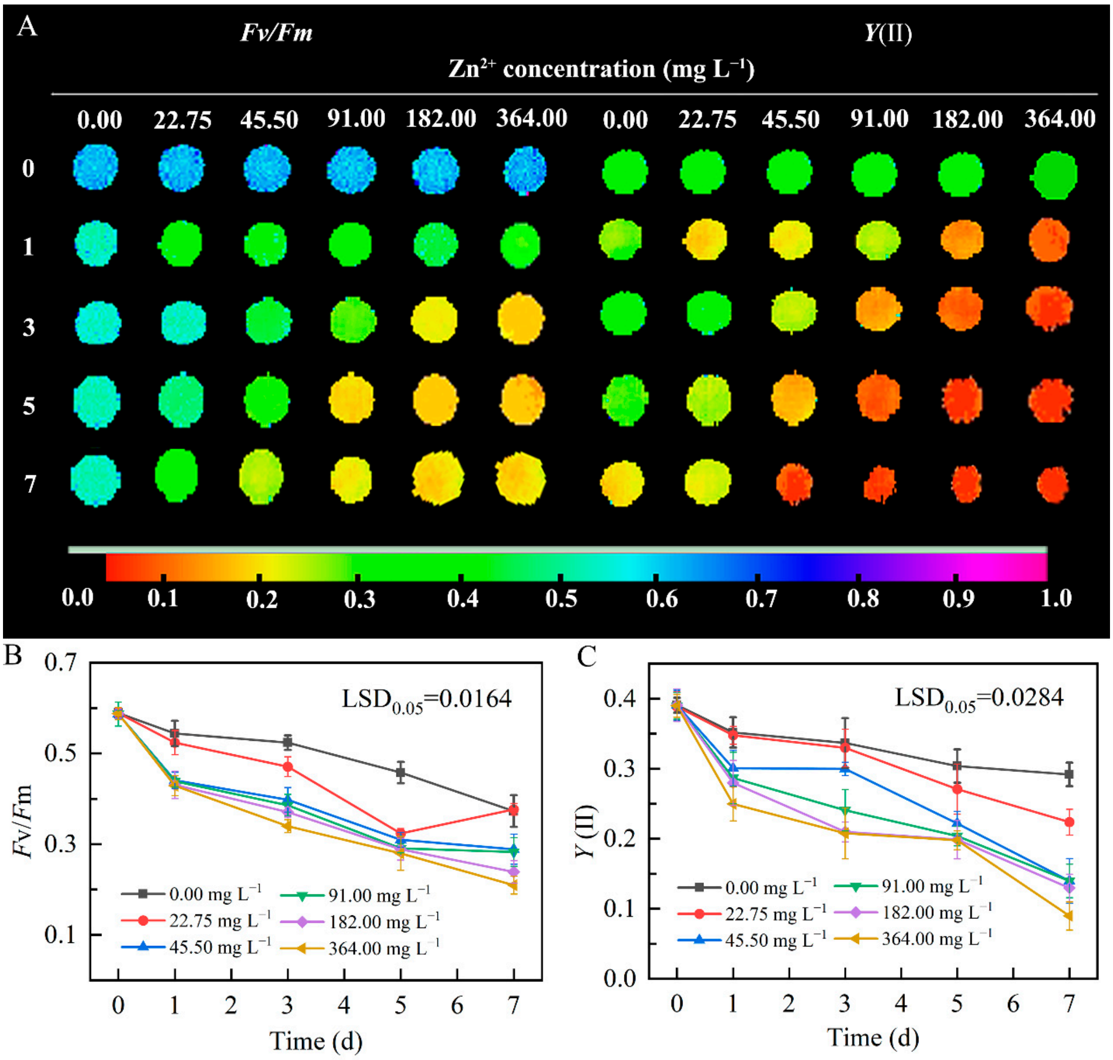

Figure S4. Zinc accumulation in *Dysmorphococcus globosus* strain ZY24 harvested after 7 days of treatment with different initial Zn^{2+} concentrations under dark conditions. Each data point represents the mean of three replicates; error bars represent the standard error of the mean.

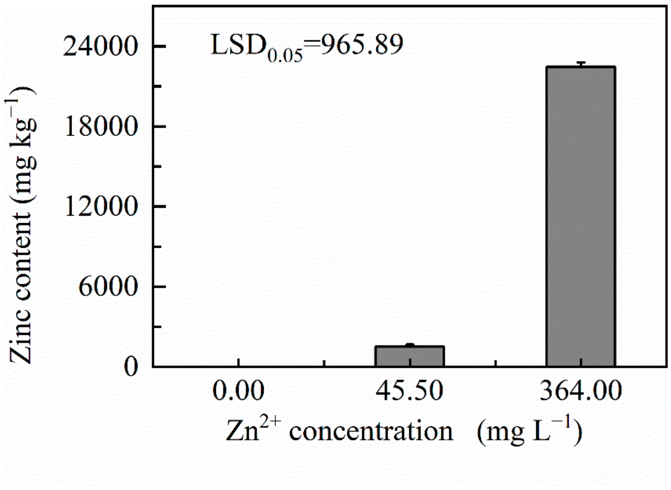

Figure S5. Liquid chromatograms of 0.00, 22.75, 45.50, 91.00, and 182.00 mg L^{-1} treatment groups on day 7, respectively. AU, absorbance unit; peak A, astaxanthin; peak B, zeaxanthin; peak C lutein; peak D, canthaxanthin; peak E, β-cryptoxanthin; peak F, β-carotene.

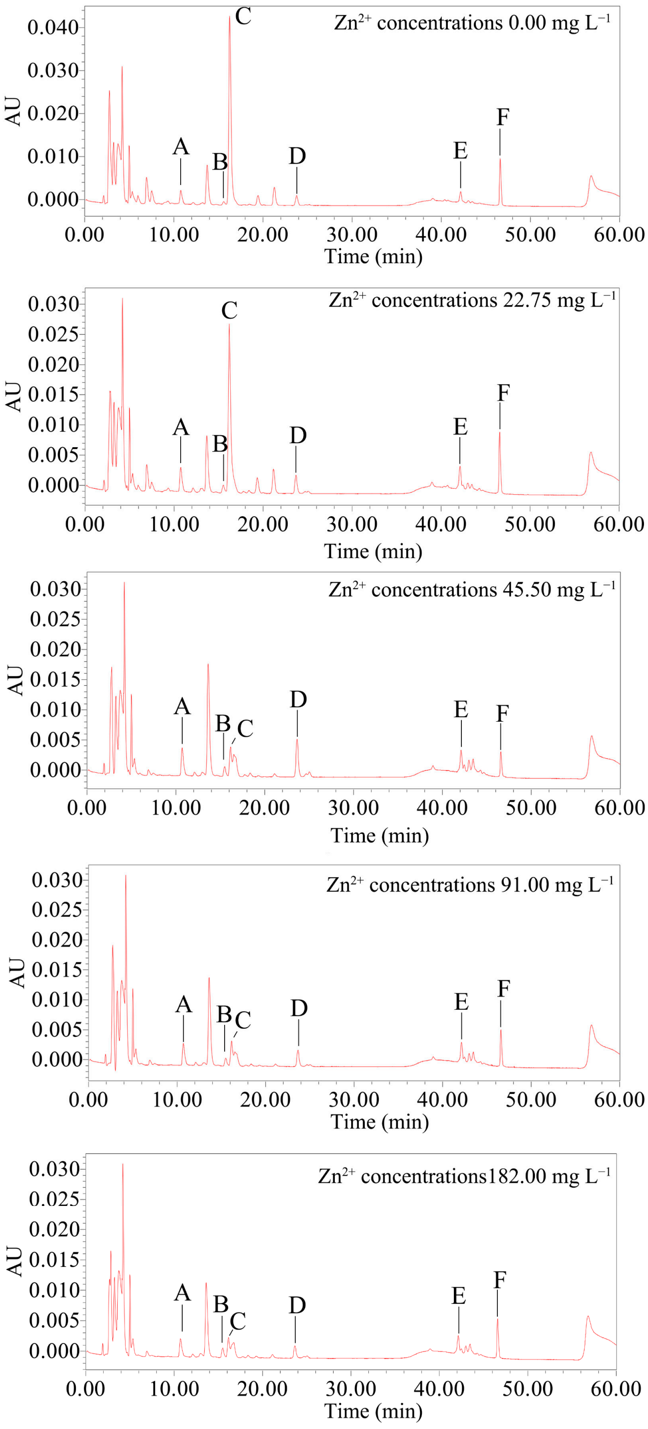

Supplement: Supplementary file 1 [file plants-13-03338-s001.zip › plants-3249071-supplementary.pdf]
